# Blockade of XCL1/Lymphotactin Ameliorates Severity of Periprosthetic Osteolysis Triggered by Polyethylene-Particles

**DOI:** 10.3389/fimmu.2020.01720

**Published:** 2020-08-04

**Authors:** Yuan Tian, Mohamad Alaa Terkawi, Tomohiro Onodera, Hend Alhasan, Gen Matsumae, Daisuke Takahashi, Masanari Hamasaki, Taku Ebata, Mahmoud Khamis Aly, Hiroaki Kida, Tomohiro Shimizu, Keita Uetsuki, Ken Kadoya, Norimasa Iwasaki

**Affiliations:** ^1^Department of Orthopedic Surgery, Faculty of Medicine and Graduate School of Medicine, Hokkaido University, Sapporo, Japan; ^2^Global Institution for Collaborative Research and Education (GI-CoRE), Frontier Research Center for Advanced Material and Life Science, Hokkaido University, Sapporo, Japan; ^3^R&D Center, Teijin Nakashima Medical Co., Ltd., Okayama, Japan

**Keywords:** periprosthetic osteolysis, XCL1, polyethylene wear debris, osteoclasts, osteoblasts

## Abstract

Periprosthetic osteolysis induced by orthopedic implant-wear particles continues to be the leading cause of arthroplasty failure in majority of patients. Release of the wear debris results in a chronic local inflammatory response typified by the recruitment of immune cells, including macrophages. The cellular mediators derived from activated macrophages favor the osteoclast-bone resorbing activity resulting in bone loss at the site of implant and loosening of the prosthetic components. Emerging evidence suggests that chemokines and their receptors are involved in the progression of periprosthetic osteolysis associated with aseptic implant loosening. In the current study, we investigated the potential role of chemokine C-motif-ligand-1 (XCL1) in the pathogenesis of inflammatory osteolysis induced by wear particles. Expressions of XCL1 and its receptor XCR1 were evident in synovial fluids and tissues surrounding hip-implants of patients undergoing revision total hip arthroplasty. Furthermore, murine calvarial osteolysis model induced by ultra-high molecular weight polyethylene (UHMWPE) particles was used to study the role of XCL1 in the development of inflammatory osteolysis. Mice received single injection of recombinant XCL1 onto the calvariae after implantation of particles exhibited significantly greater osteolytic lesions than the control mice. In contrast, blockade of XCL1 by neutralizing antibody significantly reduced bone erosion and the number of bone-resorbing mature osteoclasts induced by UHMWPE particles. In consistence with the results, transplantation of XCL1-soaked sponge onto calvariae caused osteolytic lesions coincident with excessive infiltration of inflammatory cells and osteoclasts. These results suggested that XCL1 might be involved in the development of periprosthetic osteolysis through promoting infiltration of inflammatory cells and bone resorbing-osteoclasts. Our further results demonstrated that supplementing recombinant XCL1 to cultured human monocytes stimulated with the receptor activator of nuclear factor kappa-B ligand (RANKL) promoted osteoclastogenesis and the osteoclast-bone resorbing activity. Moreover, recombinant XCL1 promoted the expression of inflammatory and osteoclastogenic factors, including IL-6, IL-8, and RANKL in human differentiated osteoblasts. Together, these results suggested the potential role of XCL1 in the pathogenesis of periprosthetic osteolysis and aseptic loosening. Our data broaden knowledge of the pathogenesis of aseptic prosthesis loosening and highlight a novel molecular target for therapeutic intervention.

## Introduction

Total joint arthroplasty (TJA) is one of the most successful medical procedures in modern clinical orthopedics, which restores the mobility of patients in the late-stage arthritis. Ensuring the long-term survivorship of TJA is significantly challenging for orthopedic surgeons because 5–10% of cases fail within 15–20 years due to the development of inflammatory osteolysis, referred to as aseptic loosening (1, 2). Loosening of the prosthesis is associated with severe pain, and poor functionality or disability and requires urgent revision surgery. Despite the health and economic impact of aseptic implant loosening on our societies, no approved therapies are currently available (1, 3). A better understanding of the molecular mechanisms underlying the occurrence of massive bone loss around the prosthesis may provide guidance for the development of therapeutic interventions.

Prosthetic particles released due to motion of the bearing surfaces of implants activates tissue-resident macrophages and triggers destructive biological responses. Upon exposure to the particles, macrophages form frustrated phagocytes which release numerous inflammatory and chemotactic mediators resulting in the activation of bone-resorbing osteoclasts leading to aseptic implant loosening (4). The magnitude of bone loss is dependent on the production of inflammatory mediators, cellular infiltration, and resorptive activity of the osteoclasts around the prosthesis (1, 3, 4). Chemokines, a group of inflammatory mediators released from the macrophages stimulated by implant particles, are known to play a role in enhancing cellular migration, production of pro-inflammatory cytokines, apoptosis, tissue remodeling, and angiogenesis in the periprosthetic tissues (2). In fact, an increase in the gene expressions of chemokines such as CCL2, CCL3, and CXCL8 is evident in periprosthetic tissues surrounding aseptically loosening implants (2). In line with this view, blocking CCL2, CXCL2, CXCL12, and their receptors has shown substantial reduction in the osteolytic activity in a murine osteolysis model, suggesting that they play crucial roles in the progression of osteolysis. These findings highlight that the interaction of chemokine-chemokine receptors is a promising therapeutic target for preventing implant loosening (2, 5, 6). Therefore, identifying of chemokines involved in the pathogenesis of periprosthetic osteolysis may provide a clue for development of new therapeutics.

Recently, the X-C motif chemokine ligand 1 (XCL1) was found to be expressed in macrophages exposed to ultra-high molecular weight polyethylene (UHMWPE) particles *in vitro* (7). XCL1, also known as lymphotactin (Ltn), is a member of the C-class containing only one of the two conserved disulfide bonds typically present in other classes of chemokines. This molecule is predominantly expressed by the T cells, synovial macrophages, fibroblast-like synoviocytes, and dendritic cells (DC) and exerts chemotactic and immunomodulatory activity on T cells, natural killer (NK) cells, and macrophages (8, 9). Nonetheless, there is a growing evidence suggesting the involvement of XCL1 in the development of arthritis and progressive bone degradation in rheumatoid arthritis (9–12). Therefore, the objective of this study was directed to investigate the possible contribution of XCL1 to the pathogenesis of periprosthetic osteolysis triggered by UHMWPE particles.

## Materials and Methods

### Immunofluorescence Staining of Synovial Tissues

Our research protocol for human samples was approved by the Research Ethics Review Committee of Hokkaido University Hospital (Approval ID: 016-0002). Informed consents were obtained from all donors for the use of samples in the research. Synovial tissues from three patients (one male of 60-years old, two females of 54- and 59-years old) undergoing revision of total hip arthroplasty were collected, fixed with 10% formalin, and embedded in paraffin. All cases were diagnosed as aseptic implant loosening and treated by doctors at Hokkaido University hospital. Sections of 3 μm size were prepared, deparaffinized, and treated for 5 min with proteinase K (Dako, CA, USA) for antigen retrieval. After blocking with horse serum for 1 h, the sections were incubated for 1 h at 37°C with primary antibody (1:500), including anti-XCR1 (R&D Systems, MN, USA) and F4/80 (Biolegend, San Diego, USA), CD68 antibody (Biolegend), or iNOS (Abcam, Cambridge, UK). The primary antibodies were detected using respective secondary antibody conjugated with Alexa Fluor^®^ 488 and Alexa Fluor^®^ 594 (Jackson ImmunoResearch, West Grove, PA, USA). The cellular nuclei were stained with DAPI (Dojindo Molecular Technologies, Kumamoto, Japan). The sections were washed with tris-buffered saline (TBS) solution, mounted, and covered using cover slips. The images were captured using a fluorescence microscope (Keyence, Osaka, Japan).

### Preparation of Polyethylene Particles

Particulate debris was generated from hip-bearing materials of ultra-high molecular weight polyethylene (UHMWPE: Teijin Nakashima medical, Okayama, Japan) (7). The materials were crushed, sterilized, and analyzed using a Multi-Beads Shocker (Yasui Kikai, Osaka, Japan), an ethylene oxide gas (EOG) sterilizer (Eogelk-SA-H160, Osaka, Japan) and the particle image analyzer Morphologi G3 (Malvern Instruments, Worcester, UK), respectively. The sizes of the prepared particulate debris were ranging between 0.1 and 100 μm. Endotoxins were detected by ToxinSensor Single Test Kit (Genscript, Piscataway, NJ, USA) according to manufacturer's instruction. Briefly, 3 mg of particles were mixed with 100 μl phosphate-buffered saline (PBS, Nacalai Tesque, Kyoto, Japan) solution and incubated for 10 min at RT. Next, the suspension was centrifugated for eliminating wear debris, mixed with LAL Reagent Water of kit, and incubated at 37°C for 1 h in a water bath. Positive reaction was characterized by the formation of firm gel. Samples were tested in in duplicates and reaction was compared to positive and negative controls. Formation of firm gel was not seen in all tested samples indicating that the endotoxin levels were below the detection limit (0.015 EU/ml). Finally, 6 ± 0.2 mg of particles were placed into Eppendorf tubes for the *in vivo* experiment.

### Murine Calvarial Osteolysis Model

Procedures for the debris-induced osteolysis model were performed as described previously (13). Briefly, 8-weeks old male C57B/6 SPF (CLEA, Tokyo, Japan) mice were injected intraperitoneally with 100 and 10 mg/kg of ketamine and xylazine, respectively, for anesthetization. A sagittal incision (~1 cm) over the calvaria anterior was made and 6 mg of UHMWPE particles were carefully implanted onto the calvarial bone. Recombinant mouse XCL1 (R&D system) at concentrations of 1 or 2 μg (dissolved in 100 μl PBS) was subcutaneously injected (single injection) onto calvariae to examine its effect on the progression of osteolysis (*n* = 7 for each group). Control mice received the UHMWPE particles and a single injection of PBS. The same surgical procedure was performed on the sham mice, but the incision was closed using stainless steel clips without further implantation of particles. In parallel experiments, mice received UHMWPE debris were injected with neutralizing mouse anti-XCL1 (Amsbio, Abingdon, UK) at dose of 5 μg on days 0 and 3 post particles implantation (*n* = 7 for each group). Control isotype antibody was used as negative control and never showed any effect *in vivo*. Mice were anesthetized with isoflurane inhalation and 100 μl antibody-diluted in PBS was subcutaneously injected onto the calvariae. For the XCL1-induced osteolysis model, collagen sponges of the single layer type (PELNAC, Tokyo, Japan) were soaked in 1 or 2 μg of recombinant mouse XCL1 (R&D system) or PBS (*n* = 6 for each group) and transplanted subcutaneously onto the calvariae for 7 days (13). All procedures for animal experiments were approved by the Institute of Animal Care and Use Committee of the Hokkaido University Graduate School of Medicine (no. 17-0085).

### Micro-CT Analysis and Bone Histomorphometry

The mice were sacrificed after 7 days and their calvariae were subjected to R-mCT2 scan analysis (Rigaku, Tokyo, Japan) for micro-computed tomography assessment (micro-CT). Bone loss on the bone surface were quantified as the percentage of bone loss surface/total area of interest using Bone J (National Institutes of Health, Washington, DC, USA) (13, 14). For conducting the histological examination, 10% of formalin-fixed calvariae were decalcified in the Decalcifying Solution B (FUJIFILM Wako Pure Chemical Corporation, Osaka, Japan) for 3 days and then embedded in paraffin. Sections of 5 μm size were prepared and subjected to staining with hematoxylin eosin (H&E, Wako), and leukocyte acid phosphatase tartrate resistance acid phosphatase (TRAP, Sigma, Tokyo, Japan). The lesions and infiltration of inflammation cells were microscopically examined using an all-in-one microscope (Keyence) and the images were analyzed with Image J (National Institutes of Health) for quantification of cells and stained area.

### Osteoclast Differentiation and Bone Resorption Assays

The osteoclasts were differentiated and cultured as described previously (13). Briefly, human primary monocytes were separated from the blood samples of healthy donors by density gradient centrifugation (Ficoll-PaqueTM PLUS: GE Healthcare, Waukesha, WI, USA) followed by treatment with a MACS Pan monocyte isolation kit (Miltenyi Biotec, Auburn, CA, USA). Our routine procedure allowed the simultaneous enrichment of classical (CD14^+^) with purity exceeding 98%. The cells were then cultured in minimum essential medium Eagle (MEM) supplemented with 10% heat-inactivated fetal bovine serum (FBS), 25 mg/l penicillin/streptomycin, and 2% L-glutamine in a 37°C-humidified atmosphere containing 5% CO_2_ in a 75 cm^2^ flask for 2 h. The adherent cells were cultured for 3 days in the same medium supplemented with 25 ng/ml of human recombinant macrophage colony-stimulating factor (MCSF, Peprotech, NJ, United States). Thereafter, cells were detached by treating with a 1% trypsin-EDTA solution (Wako) for 3 min. The 96-well culture plates or dentin slices (Wako) were seeded with 2 × 10^4^ cells/well. The cells were cultured in medium supplemented with 50 ng/ml recombinant human nuclear factor kappa B ligand (RANKL) plus different concentrations of recombinant human XCL1 (Peprotech). They were stained on day 8 using a TRAP kit (Sigma) according to the manufacturer's instructions. TRAP-positive stained cells with ≥ 3 nuclei were identified as the osteoclasts. In order to conduct cytoskeletal actin staining, the cells were fixed using 4% paraformaldehyde (Wako) for 20 min, washed with ice-cold PBS, and permeabilized using 0.1% triton X100 (Sigma) in PBS for 5 min. Cell were then stained using an Alexa Fluor 633 phalloidin (Invitrogen, Carlsbad, CA, USA). The nuclei were visualized using DAPI (Dojindo Molecular Technologies). In order to stain the bone resorption pits on slices, the cells were detached after 21 days of culture and the slices were sequentially incubated with 20 mg/ml of peroxidase-conjugated wheat germ agglutinin and 3,3'-diaminobendzidine (0.52 mg/mL in PBS containing 0.1% H_2_O_2_). Resorption pits were visualized using a confocal microscope and measured as the percentage of resorbed bone surface per total bone surface area using an image analysis software (ImageJ, National Institutes of Health). All experiments were performed at least twice in triplicate wells/slices for reproducibility of data.

### Osteoblast Differentiation and Cultivation

Human primary fetal osteoblasts (hFOB) purchased from Cell Applications (San Diego, CA, USA) were allowed to proliferate up to four passages in an osteoblast growth medium (Cell Applications). The cells were detached using a solution of 1% trypsin-EDTA (Wako) and cultured either in 96-well or 24-well plates using an osteoblast differentiation medium (Cell Applications) supplemented with or without 50 or 100 ng/ml of recombinant XCL1 or tumor necrosis factor-α (TNF-α, Peprotech) for 21 days. The cultures were regularly replenished with fresh media every 3 days. Thereafter, the cells in the 96-well plates were washed with PBS containing MgCl2 and CaCl2 (Sigma), fixed using a 10% formalin neutral buffer solution (Wako), and stained either with alizarin red-S or a BCIP/NBT solution (Wako). The optical density (OD) values were read at 490 nm using a Benchmark Plus Microplate Spectrophotometer System (Bio-Rad, Tokyo, Japan). In a separate experiment, differentiated hFOB (1 × 10^5^) were cultured in differentiation medium (Cell Applications) and stimulated with either recombinant human XCL1 or TNF-α (Peprotech) at concentration of 50 ng/ml for 48 h.

### Human Fibroblast-Like Synoviocytes (HFLS) Cultivation and Activation

HFLS from normal healthy human synovial tissues purchased from Cell Applications (Cell Applications) were cultured in full growth medium according to the supplier's recommendations. All experiments were conducted on cells from passage 3. Cells were seeded (2 × 10^4^/well), incubated in basal medium (Cell Applications) overnight, and then stimulated with either recombinant XCL1 at concentration of 10, 50, or 100 ng/ml or TNF-α (Peprotech) at concentration of 10 ng/ml for 48 h.

### Quantitative Real-Time Polymerase Chain Reaction (qRT–PCR)

Cells and homogenized tissues were lysed using TRIzol Reagent (Invitrogen) for RNA extraction. To obtain RNA from calvarial bones following implantation of wear particles, frozen samples were crushed using tissue homogenizer (Power Masher II, Nippi, Tokyo, Japan) in liquid nitrogen. Cells (1 × 10^6^) from the granulomatous tissue around UHMWPE particles were obtained on day 7 and then digested by trypsin solution (Wako) for 10 min in 37°C-water bath. Thereafter, chloroform (Wako) was added for phase separation and the DNA-free RNA was purified from the aqueous layer using RNeasy Plus Mini kit columns (Qiagen, Hilden, Germany). Purified RNA (0.5 μg) was reverse transcribed using the GoScriptTM reverse transcriptase kit (Promega, Madison, USA) in order to carry out a qRT-PCR analysis. The cDNAs were assayed using the SYBR^®^ Premix Ex Taq™ II (Takara, Shiga, Japan) and gene-specific primers listed in Supplementary Table (13). Specific primers were designed using Primer-BLAST (https://www.ncbi.nlm.nih.gov/tools/primer-blast/) to amplify 60–100 bp of the genes. The gene expression was calculated by the ^2−ΔΔ^Ct method after normalizing to the expression of the housekeeping genes, including GAPDH and β-actin. Amplification efficiencies of the target and reference genes ranged between 90 and 110%.

### Western Blotting

Cells were lysed on ice in RIPA lysis buffer (ATTO corporation, Tokyo, Japan). Proteins were separated in SDS-PAGE gels by electrophoresis and then transferred to polyvinylidene fluoride membrane (Immobilon-P Membrane; Merck, Darmstadt, Germany). Primary antibodies were against of β-actin, nuclear factor-κB (NF-κB) P65 (Biolegend) and mitogen-activated protein kinases (MAPKs) of P38, phosphor-P38, ERK1/2, and SAPK/JNK (Cell signaling technology, MA, USA). HRP-conjugated anti-rabbit or anti-goat antibodies (Novus Biologicals) were used as secondary antibodies. The bands were visualized using a Quantity One v. 4.6.9 (Bio-Rad) software and quantification of intensity were carried out using ImageJ software (National Institutes of Health). Relative intensity of each band was calculated based on bands intensity of target/ β-actin.

### Statistical Analysis

One-way analysis of variance (ANOVA) followed by Tukey's multiple-comparison procedure was used to compare differences among groups and Student's *t*-test was used to compare the differences between two independent groups (GraphPad Software, La Jolla, CA, USA). Results presented as means ± standard errors of the means (SEM) were considered statistically significant when *p* < 0.05. The gene expressions in calvarial bone tissue of mice that received XCL1-soaked sponges and the PBS-soaked sponges were presented by heat map (GraphPad).

## Results

### Upregulation of XCL1 and XCR1 in Periprosthetic Tissues Around Implant Particles

**Figure 1:**
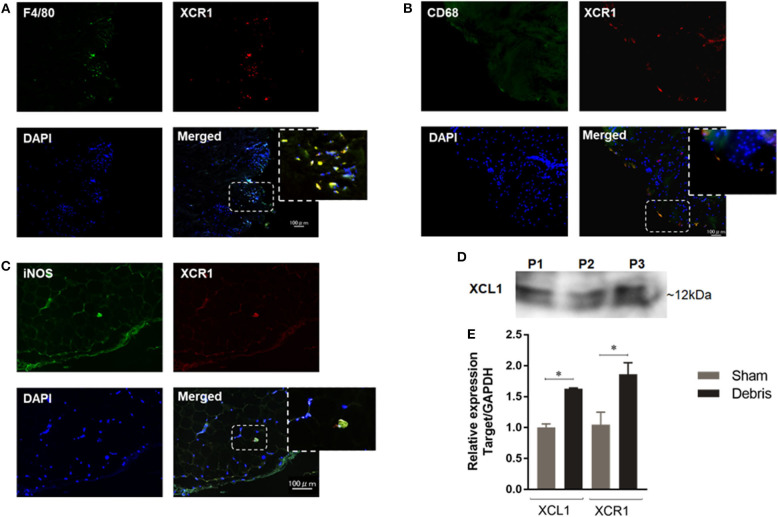
Expression of XCL1 and XCR1 in periprosthetic tissues. **(A–C)** Detection of XCR1 in human tissues around loosening hip-implant by the immunofluorescence test. Deparaffinized sections were stained to observe F4/80, CD68, iNOS (green), XCR1 (red), and cell nuclei (blue). Scale bars are 100 μm. Representative images exhibit the sectioned tissues from three patients. **(D)** Detection of XCL1 in synovial fluid from same patients by Western blotting analysis. **(E)** Gene expressions of XCL1 and XCR 1 in calvarial bone tissues in a murine osteolysis calvarial model. Results represent the means of relative expression values ± SEM of three mice. ^*^indicates a significant difference as determined by the Student *t*-test (*p* ≤ 0.05).

In order to assess the involvement of XCL1 in the inflammatory osteolysis induced by polyethylene particles, synovial tissues around loosening hip-implant were sectioned and immunostained with specific antibodies to XCR1. Positive signals representing XCR1 expression were observed in cells filling the synovial tissues from patients undergoing revision surgery (Figure 1A). It is significant to note that majority of XCR1-positive cells were co-stained with the F4/80, CD68, and iNOS antibodies used as macrophages/dendritic cells markers (Figures 1A–C). Consistent with these results, XCL1 was detected in synovial fluids collected from the same patients (Figure 1D). To confirm whether the XCR1 and XCL1 is upregulated in the periprosthetic tissues, we employed wear particles-induced inflammatory osteolysis of murine model. UHMWPE particles were implanted onto calvaria and gene expression of XCL1 and XCR1 was examined in calvarial bone. Both genes were significantly upregulated (*p* = 0.0004 and 0.0414, respectively) in mice with the implanted UHMWPE wear particles compared to the control (sham) mice (Figure 1E). From these results, we inferred that XCL1 is upregulated in periprosthetic tissues from patients undergoing revision total hip arthroplasty due to the presence of implant particles around loosening implant.

### XCL1 Is Involved in Progression of Inflammatory Osteolysis in Murine Model

**Figure 2:**
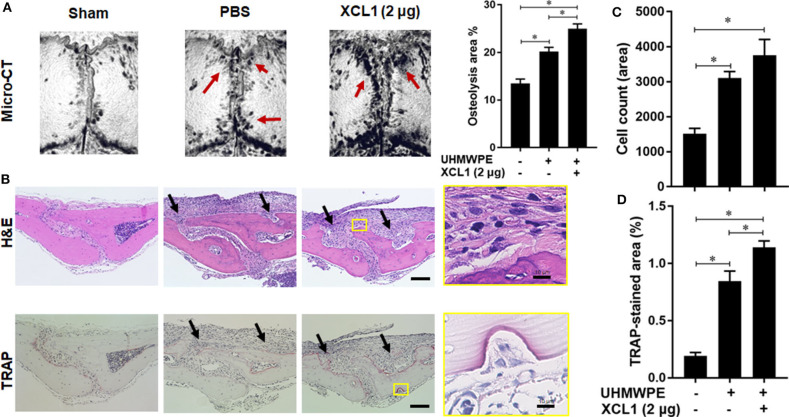
Administration of XCL1 exaggerates osteolytic lesions in a polyethylene-particles-induced osteolysis model. **(A)** Representative images for micro-CT of calvariae. The right panel shows quantification of the lytic area on the calvarial bone tissues of mice. Results represent the means ± SEM of seven mice. ^*^represents the significance determined by one-way ANOVA, followed by a Tukey's multiple-comparison procedure. Arrows indicate osteolytic lesions. **(B)** Representative images for histological analyses of bone sections stained by H&E and TRAP. Scale bar is 100 μm. Arrows indicate bone lesions. **(C)** Cell count of inflammatory cells in calvarial bone sections. **(D)** Quantification of TRAP-stained areas in calvarial bone sections. The results represent the means ± SEM for three mice. ^*^indicates a significant difference, as determined by one-way ANOVA, followed by the Tukey's multiple-comparison procedure (*p* ≤ 0.05).

**Figure 3:**
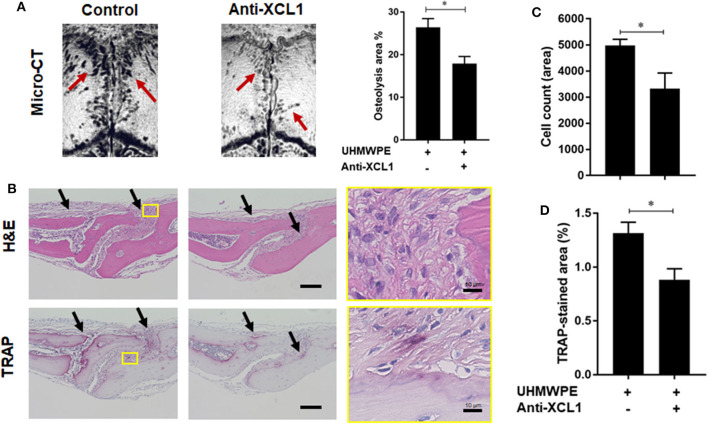
Blockade of XCL1/lymphotactin by neutralizing antibody ameliorates severity of osteolysis triggered by polyethylene-particles in murine model. **(A)** Quantification of the lytic area on the calvarial bone tissues of mice determined by micro-CT. Left panel shows representative images of calvariae. Results represent the means ± SEM of seven mice. ^*^represents the significance determined by one-way ANOVA, followed by a Tukey's multiple-comparison procedure. Arrows indicate osteolytic lesions. **(B)** Histological analyses of bone sections stained by H&E and TRAP. Scale bar is 100 μm. **(C)** Cell count of inflammatory cells in calvarial bone sections. **(D)** Quantification of TRAP-stained areas in calvarial bone sections. Arrows indicate bone lesions. The results represent the means ± SEM for four mice. ^*^indicates a significant difference, as determined the Student *t*-test (*p* ≤ 0.05).

Detection of XCL1 in periprosthetic tissues encouraged us to speculate that it may have a role in the progression of inflammatory osteolysis and aseptic loosening. To examine our speculation, UHMWPE particles were implanted onto the mice calvariae followed by a single injection of recombinant murine XCL1 (2 μg = 0.1 mg/kg), and the bone lesions were histomorphometrically assayed after 7 days. It was observed that mice receiving 2 μg XCL1 exhibited significantly greater osteolytic lesions (*p* = 0.0297) than the control mice, which were implanted with the UHMWPE particles and injected with PBS (Figure 2A). Histologically, XCL1-injected mice showed larger areas of bone loss and greater infiltration of inflammatory cells (*p* =0.0038) as well as TRAP-stained area (*p* = 0.0363) than control mice (Figures 2B–D). Monocytic inflammatory cells (F4/80^+^/iNOS^+^) were detected in the cell-infiltrated area (Supplementary Figure 1). These results revealed that XCL1 might contribute to the osteolytic activity triggered by the UHMWPE particles *in vivo*. To confirm our findings, neutralizing antibody to XCL1 was used to functionally block this molecule in calvarial murine model. UHMWPE particles were implanted onto the calvariae followed by the two injections of 5 μg mouse XCL1 neutralizing antibody onto calvariae on day 0 and 3. Micro-CT analysis revealed that neutralizing XCL1 by antibody resulted in significant reduction (*p* = 0.0076) in sizes of osteolytic lesions (Figure 3A). Moreover, these mice showed lesser osteolytic lesions with a reduced infiltration of inflammatory cells (*p* = 0.0431) and TRAP-stained area (*p* = 0.0230) in calvarial bones (Figures 3B–D). These results demonstrated that blocking of XCL1 significantly reduced bone erosion induced by implant wear particles in murine calvarial model. To further gain an insight into the role of XCL1 in the development of local inflammatory cytokines, cells from granulomatous tissue formed around UHMWPE particles in mice were separated and subjected for gene expression analysis. In consistent with the above results, treatment with XCL1 neutralizing antibody reduced the gene expression of inflammatory (40% for IL-6 and 10% for TNF-α) and osteoclastogenic factors (30% for TRAP, 25% for OSCAR, and 20% for CTSK) in granulomatous tissue (Supplementary Figure 2). These findings indicated that presence of XCL1 in periprosthetic tissues might promote the infiltration of inflammatory cells and production of inflammatory and osteoclastogenic factors. Together, our results revealed that XCL1 might be involved in development of periprosthetic osteolysis leading to aseptic loosening.

### Transplantation of XCL1-Soaked Sponges Induces Local Bone Erosion

**Figure 4:**
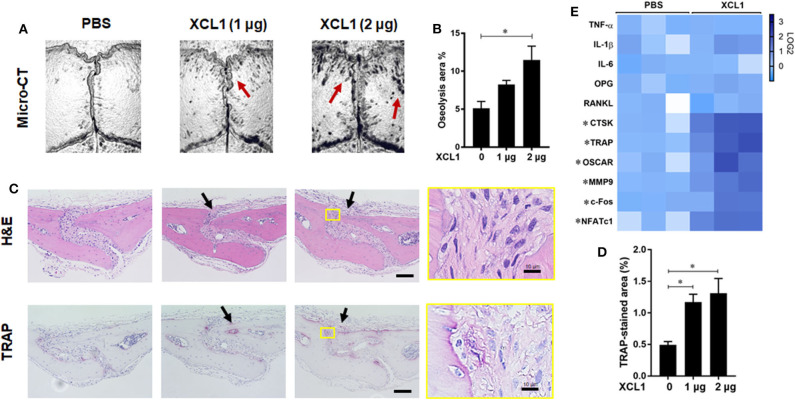
Sponge-soaked protein-induced murine model. **(A)** Representative images for micro-CT of calvariae. Arrows indicate osteolytic lesions. **(B)** Quantification of the lytic area on the calvarial bone tissues of mice determined by micro-CT. Results represent the means ± SEM of six mice. ^*^represents the significance determined by one-way ANOVA, followed by a Tukey's multiple-comparison procedure. **(C)** Representative images for histological analyses of bone sections stained by H&E and TRAP. Scale bar is 100 μm. Arrows indicate bone lesions. **(D)** Quantification of TRAP-stained areas in calvarial bone sections. Results represent the means ± SEM of three mice. ^*^indicates a significant difference, as determined by one-way ANOVA, followed by the Tukey's multiple-comparison procedure (*p* ≤ 0.05). **(E)** Heat map for the gene expression of inflammatory and osteoclast marker genes in bone tissues. Calvarial bone tissues were harvested for the analysis of gene expressions after the implantation of XCL1-soaked sponges. Scale bar (Log2) represents the means of relative expression values of each target gene after normalizing with the GAPDH ± SEM of three mice. ^*^indicates a significant difference, as determined by the *t*-test (*p* ≤ 0.05).

To further gain direct evidence on the involvement of XCL1 in the development of bone lesions, chemokine-soaked sponges were transplanted onto the calvariae and histomorphometric analyses were preformed after 7 days. It was noted that the transplantation of 2 μg XCL1-soaked sponges induced local bone loss as detected by micro-CT (*p* = 0.0053) (Figures 4A,B). Histological observations revealed a greater infiltration of inflammatory cells and larger TRAP-stained areas (PBS vs. XCL1 1 μg *p* = 0.0500, PBS vs. XCL1 2 μg *p* = 0.0234) compared to those of mice that received PBS-soaked sponges (Figures 4C,D). Likewise, greater TRAP-positive areas were seen in the whole skulls of these mice (Supplementary Figure 3). In the cell-infiltrated area, few F4/80^+^/iNOS^+^ cells as monocytic inflammatory cells were detected (Supplementary Figure 1), indicating the development of inflammation. Furthermore, we examined the gene expression of inflammatory cytokines and osteoclast markers in the calvarial bone tissues collected after 4 days of sponge transplantation. The gene expressions of CTSK, TRAP, OSCAR, MMP9, c-Fos, and nuclear factor of activated T-cells, cytoplasmic 1 (NFATc1) were significantly elevated (*p* < 0.05) in mice that received XCL1-soaked sponges compared to those with the PBS-soaked sponges (Figure 4E). Collectively, our data suggested that XCL1 can promote inflammation and bone resorption *in vivo*.

### XCL1 Promotes Osteoclasts Differentiation and Bone Resorption Activity

**Figure 5:**
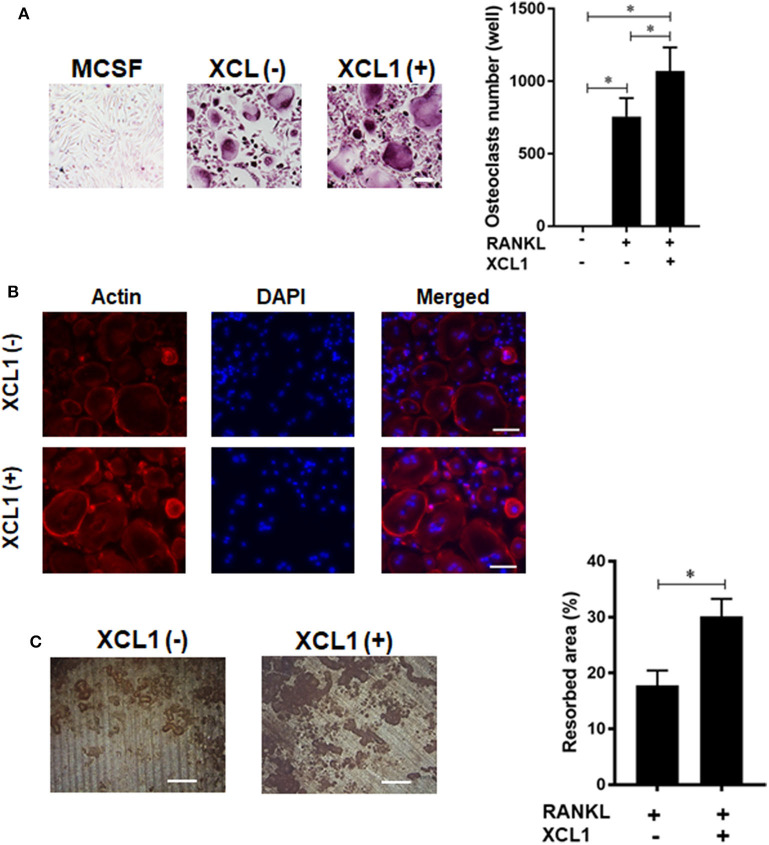
Effect of XCL1 on osteoclast differentiation and bone resorption. **(A)** Count of TRAP-positive cells in RANKL-stimulated monocytes in the presence or absence of XCL1. Left panel shows representative images for cells stained by TRAP. Results represent the means ± SEM of triplicates. ^*^indicates a significant difference, as determined by the Tukey's multiple comparisons test (*p* ≤ 0.05). **(B)** Actin ring staining assay for cells in RANKL-stimulated monocytes in the presence or absence of XCL1. **(C)** Quantification of the bone resorbed areas on dentin slices. Results represent the means ± SEM of values from three dentin slices. Left panel shows representative images for the resorbed areas. Scale bars are 200 μm.

Given the central role of osteoclasts in development of inflammatory osteolysis, we examined the effect of recombinant human XCL1 on the differentiation and function of osteoclasts *in vitro*. Addition of XCL1 to the RANKL-stimulated monocytes (50 ng/ml) resulted in a significant elevation in the number of TRAP-positive cells (*p* = 0.0419) (Figure 5A), (Supplementary Figure 4). Bearing in mind that cytoskeletal rearrangements and actin ring formation are essential for bone resorption activity of osteoclasts, we stained actin ring formation with rhodamine-conjugated phalloidin. Osteoclasts treated with XCL1 displayed more dense and prominent actin rings compared to osteoclasts generated by RANKL alone (Figure 5B). In consistence with these results, bone resorption assay showed that the addition of XCL1 to RANKL-stimulated monocytes resulted in significantly larger pits on dentin slices (Figure 5C). TRAP-positive cells and pits were not detected in cultures of monocytes stimulated with XCL1 alone (data not shown). Gene expressions of osteoclast differentiation markers including TRAP, OSCAR, NFATc1, and CTSK were slightly elevated in cells treated with XCL1 (Supplementary Figure 5). Together, these results revealed that XCL1 exerts a synergistic effect along with RANKL that promotes osteoclast differentiation and bone resorption activity.

### XCL1 Promotes the Expression of Inflammatory and Osteoclastogenic Factors in Differentiated Osteoblasts but Not in FLS

**Figure 6:**
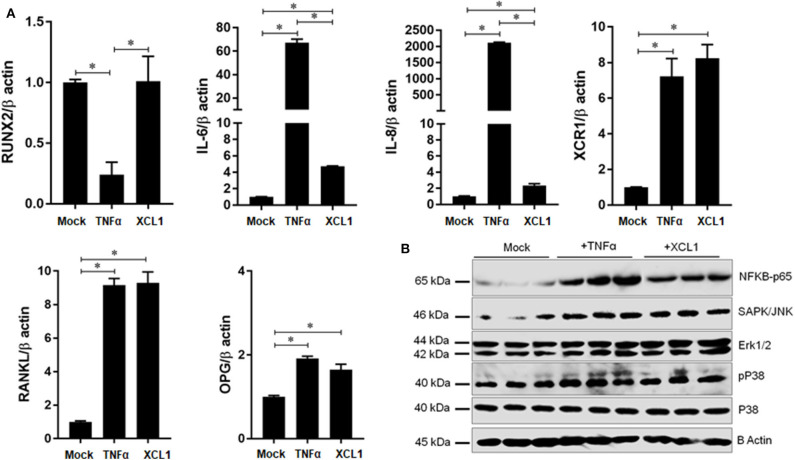
Effect of XCL1 on osteoblast activation and function. **(A)** Effect of XCL1 on the gene expression of inflammatory and osteoclastogenic factors in differentiated osteoblasts. Results represent the means ± SEM of triplicates and ^*^indicates a significant difference, as determined by the Tukey's multiple comparisons test (*p* ≤ 0.05). **(B)** Effects of stimulation by recombinant proteins on NFKB, SAPK/JNK, P42/p44-MAPK (Erk1/2), and P38- activities. Human osteoblasts were cultured in a differentiation medium supplemented with either XCL1 or TNFα (positive control) and harvested for the gene expression analysis by qRT-PCR or for protein analysis by western blotting.

To gain a new evidence that XCL1 is involved in the development of periprosthetic osteolysis, we studied its effects on osteoblast differentiation and expression of inflammatory and osteoclastogenic factors. Recombinant human TNF-α was used as positive control to verify the condition of our experiment. Cells were cultured in osteoblast differentiation medium for 21 days in presence or absence of stimuli and then stained by alizarin red-S and BCIP/NBT for quantifying calcium deposits and alkaline phosphatase activity, respectively. Staining revealed that recombinant XCL1 at 50 ng/ml exhibited no negative effects on the differentiation of osteoblasts (Supplementary Figure 6). Consistently, osteoblasts stimulated by XCL1 for 48 h showed no significant changes in gene expression of runt-related transcription factor 2 (RUNX2) known as key transcription factor associated with osteoblast differentiation (Figure 6A). In contrast, there was a significant elevation in the expression of inflammatory and osteoclastogenic factors, including IL-6, IL-8, OPG, and RANKL as compared to control osteoblasts (mock) (Figure 6A). In addition, an increase in the expression of XCR1 was noted in osteoblasts stimulated with either XCL1 or TNF-α (Figure 6A), which may suggest the involvement of XCL1 and XCR1 in activation of osteoblasts. In consistent with these results, expression of NF-κB P65 and phosphor-P38, ERK1/2 and SAPK/JNK was increased in stimulated osteoblasts (Figure 6B), (Supplementary Figure 7). These results suggested that XCL1 may promote inflammatory osteolysis through activation of osteoblasts to express both inflammatory and osteoclastogenic factors. Bearing in mind that fibroblasts are the dominant cell type in the interface membrane and play a role in the pathogenesis of periprosthetic osteolysis associated with particulate wear debris (15), we examined the effects of recombinant XCL1 on the expression of inflammatory and osteoclastogenesis factors in HFLS. Notably, there were no significant increases in the expression of IL-8, IL-6, TNFα, CCL20, MMP3, and MMP1 in cells stimulated with XCL1 (Supplementary Figure 8). Likewise, differentiated macrophages stimulated with XCL1 for 24 h didn't show a significant increase in gene expressions of TNF-α, IL-1β, IL-6, IL-8, CCL5, CCL20, CXCR3 (data not shown). From these results, we inferred that XCL1 exerts immunomodulatory activity on specific cells in the tissues.

## Discussion

The inability to develop an effective therapeutic agent for the prevention of aseptic loosening is probably due to the lack of knowledge on the pathogenesis of inflammatory osteolysis (1, 2). However, pathologic bone resorption mediated by osteoclasts following inflammation triggered by implant wear particles is the principal cause of aseptic loosening and arthroplasty failure (2, 3). Therefore, exploring the molecular mechanisms mediating the activation of osteoclasts may aid in developing new therapeutic agents for this health problem.

Chemokines play a significant role in the development of inflammation around orthopedic implants through enhancing cells migration, growth and differentiation (16). Activated macrophages by wear particles are believed to be the major source of chemokines in periprosthetic tissues (17). Of these chemokines, XCL1 that has been reported to be upregulated in macrophages stimulated with UHMWPE wear debris *in vitro* (7). XCL1 is expressed by numerous immune cells, including activated T cells, NK cells, and thymic medullary epithelial cells. The chemotactic function of XCL1 is dependent on its binding to XCR1 expressed in the antigen presenting cells resulting in recruitment of inflammatory cells. Our current data confirmed the expression of XCL1 and its receptor XCR1 in the tissues around loosening hip-implants suggesting the potential involvement of the XCL1 and XCR1 in the development of inflammatory osteolysis and aseptic loosening. In line with this view, XCL1 has been detected in synovial fluid, and XCR1 have been detected in the infiltrated mononuclear cells and synoviocytes within synovial tissues of rheumatoid arthritis patients (9). These findings suggest that XCL1 and XCR1 play a pathological role in joint diseases. In fact, the pathological role of XCL1-XCR1 axis has been implicated in numerous chronic inflammatory diseases, including rheumatoid arthritis, inflammatory arthritis, Crohn's disease, acute allograft rejection, autoimmune disorders, encephalomyelitis, crescentic glomerulonephritis, and inflammatory bowel disease (18–23).

To further understand the role of XCL1 in the development of periprosthetic osteolysis riggered by implant debris, recombinant murine XCL1, and function-blocking antibody were used in UHMWPE particle-induced osteolysis murine model. Results revealed that administration of recombinant XCL1 exacerbated the osteolytic activity triggered by UHMWPE wear particles in a murine calvarial model. Histologically, the lytic bone lesions were occupied by excessive infiltrates of the inflammatory cells and osteoclasts. On the other hand, blocking XCL1 following the implantation of UHMWPE particles ameliorated bone erosion and recruitment of inflammatory cells in calvarial bone tissues. These results are consistent with earlier study showed that administration of recombinant XCL1 results in exacerbated inflammatory arthritis typified by severe erosion of the joint cartilage, infiltration of inflammatory cells, and extensive destruction of proteoglycans in mouse model. Furthermore, authors showed that neutralization of XCL1 by a specific anti-XCL1 monoclonal antibody ameliorates the severity of inflammatory arthritis in these mice (11). Together, our findings suggest that XCL1-XCR1 axis may be an attractive therapeutic target for inflammatory osteolysis induced by wear particles through suppression of cellular infiltrates and bone resorption in periprosthetic tissues. These results support the concept that blocking the chemokine-receptor axis may offer a promising and innovative approach for attenuating the progression of diseases with bone resorption (2). In line with this view, treatment with the CCL9 antibody suppresses osteoclastogenesis *in vitro* and *in vivo* rat model (24). Likewise, blockage of CXCR2 attenuates osteoclast formation and osteolysis induced by the titanium particle model (5).

Our further results showed that XCL1 promoted osteoclasts differentiation and bone resorption *in vitro*. The findings can be explained by the fact that chemokines are able to promote migration and fusion of the monocyte lineage *in vitro* through increasing the of integrin α9β1(2). Another possibility to explain the promoted osteoclastogenesis in our study might be that activation of XCL1-XCR1 axis initiates PI3K/AKT, MEK, and JNK signaling pathways in a variety of cells (25, 26). These signaling pathways are involved in regulation of osteoclasts differentiation and bone resorption activity. These results agreed with earlier findings highlighting the involvement of chemokines including CCL3, CCL5, CCL9, CCL19, CCL20, CCL21, and CX3CL1 in the differentiation of osteoclasts (2, 27–30). Together, it is most likely that all classes of chemokines may act as potent osteoclastogenic factors promoting homing and maturation of osteoclasts.

There are over 40 distinct chemokines clustered into four families according to the sequentially conserved cysteine residues at their N-termini. The CC chemokines have two adjacent cysteines, while a single amino acid separating the cysteines in CXC chemokine class, and three intervening amino acids are between the cysteine residues in CX3C chemokines. X-C chemokines are unique class of chemokines that have one of the two cysteine residues (16). Several lines of evidences suggest that the chemotactic activities of chemokines are crucial for bone physiology and pathophysiology in various bone diseases through orchestrating cellular homing and osteoclastogenesis (31). Osteoclasts are multinucleated giant cells derived from the monocyte-macrophage lineage and their survival and differentiation are essentially dependent on the activation caused by RANKL and MCSF. Stimulation of macrophages by RANKL activates RANK, which recruits TNF-receptor associated factors (TRAFs) and initiates several signaling pathways including MAPKs and NF-κB. This leads to activation of c-Fos and NFATc1, which are crucial for terminal osteoclast differentiation. Mature osteoclasts express specific genes, including those for TRAP, CTSK, and MMP9 that play an essential role in carrying out their function (2). Moreover, activation of αvβ3 integrin, tyrosine kinase Syk, and proto-oncogene c-Src is an essential signaling complex in osteoclasts for bone resorption process. Therefore, the role of chemokines system in enhancing differentiation of osteoclasts might rely on their ability to activate MAPKs and NF-κB signaling pathways, and other positive regulators of osteoclastogenesis such as integrins, c-Fos, and NFATc1 (32).

Another important finding in our study is that stimulation by recombinant XCL1 increased the expression of RANKL in human osteoblasts. These results revealed the involvement of XCL1 and XCR1 in the activation of osteoblast-mediated osteoclastogenesis associated with bone resorption. Therefore, it might be interesting to further explore the precise mechanism by which XCL1 activates osteoblasts. In line with this view, other chemokines such as CXCL8, CCL20, and CXCL5 have been documented to promote osteoclastogenesis through mediating the production of RANKL expression and inflammatory cytokines (33, 34). It is worth mentioning that recombinant XCL1 did not upregulate inflammatory molecules in fibroblasts and macrophages, which may suggest that XCL1 has selective action on the cells. These results are consistent with earlier findings noted that XCL1 can selectively activate DCs and CD8+ T cells. In fact, XCL1 is an integral for development of efficient cytotoxic immunity *in vivo* through enhancing the capacity of CD8+ T cells to secrete interferon gamma (IFN-γ) (35). Taken together, blocking of the XCL1-XCR1 axis may be a potent therapeutic target for preventing aseptic loosening through the suppression of cellular infiltrates, inflammation, and differentiation of osteoclasts at the site of implant. The pivotal role of XCL1 in activation of osteoclasts and osteoblasts highlights it as a therapeutic target for aseptic loosening and other diseases associated with bone destruction such as rheumatoid arthritis, Paget's disease, cancer bone metastases, and osteoporosis.

**Figure 7:**
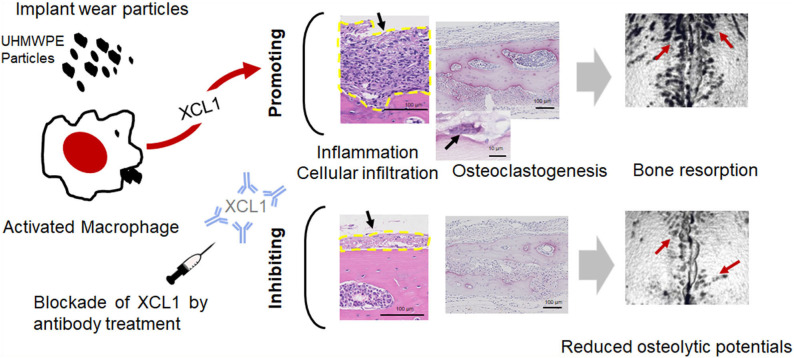
The summary of the current study. XCL1 promotes cells infiltrate, inflammatory response, and osteoclast differentiation leading to aseptic loosening. Scale bars are indicated on histological images. Blocking of the XCL1 might be a potent therapeutic target for this clinical problem.

In conclusion, our results provide an evidence on the potential role of XCL1 and XCR1 in the progression of inflammatory osteolysis and aseptic loosening. XCL1 seemed to play a dual role in inflammatory osteolysis serving as a potent chemoattractant for inflammatory cells and osteoclastogenic factor causing extensive bone destruction (Figure 7). Our data highlight XCL1 as a new molecular target for therapeutic intervention for inflammatory osteolysis and aseptic implant loosening. Our further study includes the use of RANKL blocking antibody with XCL1 antibody for treatment of pathological bone loss in murine models.

## Data Availability Statement

The raw data supporting the conclusions of this article will be made available by the authors, without undue reservation, to any qualified researcher.

## Ethics Statement

The studies involving human participants were reviewed and approved by The Research Ethics Review Committee of Hokkaido University Hospital (Approval ID: 016-0002). The patients/participants provided their written informed consent to participate in this study. The animal study was reviewed and approved by the Institute of Animal Care and Use Committee of the Hokkaido University Graduate School of Medicine (no. 17-0085).

## Author Contributions

Study designs were done by YT, MT, and TO. *In vivo* and *in vitro* experiments were performed by YT, HA, GM, MH, KU, and TE. Data acquisition and statistical analysis were done by YT, MT, DT, and KK. Drafting of manuscript was performed by YT and MT. Critical review of manuscript was done by NI, TO, MA, and HK. All authors have read and approved the submitted manuscript.

## Conflict of Interest

KU is an employee of Teijin Nakashima Medical the producer of UHMWPE-based implants. The remaining authors declare that the research was conducted in the absence of any commercial or financial relationships that could be construed as a potential conflict of interest.
