# Supplementary material for: Blockade of XCL1/Lymphotactin Ameliorates Severity of Periprosthetic Osteolysis Triggered by Polyethylene-Particles
Source: Front Immunol. 2020 Aug 4;11:1720. doi: 10.3389/fimmu.2020.01720 (PMC7417302; doi:10.3389/fimmu.2020.01720)
Supplement: Supplementary file 1 [file Data_Sheet_1.docx]

Supplementary Material

# Supplementary Figures and Tables

## Supplementary Table

**Supplementary Table.** Primers used in this study for qRT-PCR.

| **Target** | **Forward** | **Reverse** |
| --- | --- | --- |
| **mIL-1β** | GCTTCAGGCAGGCAGTATCA | AAGGTCCACGGGAAAGACAC |
| **mIL-6** | TAGTCCTTCCTACCCCAATTTCC | TTGGTCCTTAGCCACTCCTTC |
| **mIL-11** | AACTAGCTGCACAGATGAGAGA | AAGATCCCAATGTCCCAGCG |
| **mSOFAT** | TTGCTCAGAAGATGGGCCTG | GATGTCGTTGCGGTTCACTG |
| **mIL-10** | AAGGGTTACTTGGGTTGCCA | CCTGGGGCATCACTTCTACC |
| **mTGF-β** | TGGAGCTGGTGAAACGGAAG | CTGGCGAGCCTTAGTTTGGA |
| **mOPG** | TTTGCCTGGGACCAAAGTGA | TTGTGAGCTGTGTCTCCGTT |
| **mRANKL** | AGGGAGCACGAAAAACTGGT | CGGAGCTTGAAAAATCCCCC |
| **mTRAP** | TCTTCAGGACGAGAACGGTG | CCTTTCGTTGATGTCGCACA |
| **mOSCAR** | ACTCTCTGTGAGCTGTCCCTC | TGGGGGTATGAGGCTAGGGG |
| **mMMP9** | GCCAGCCGACTTTTGTGGTC | GTGTCCGTGAGGTTGGAGGT |
| **mDCSTAMP** | CAAGGAACCCAAGGAGTCGT | AGGAATGCAGCTCGGTTCAA |
| **mc-Fos** | TACTACCATTCCCCAGCCGA | CGGACAGATCTGCGCAAAAG |
| **mNFATc1** | GGAGAGTCCGAGAATCGAGAT | TTGCAGCTAGGAAGTACGTACGTCT |
| **mTNFAIP6** | ATACAAGCTCACCTACGCCG | TTGTAGGTTGCGAGACGACC |
| **mITGA9** | GGCTCGTTTTATTGGGCTGG | TTGTCCGTGAGGTTCAGCAC |
| **mGAPDH** | TGCAGCGAACTTTATTGATG | ACTTTGTCAAGCTCATTTCC |
| **hRUNX2** | TCTCCAGGAGGACAGCAAGA | GCAGCCTTAAATGACTCTGTTGG |
| **hOPG** | CGTGTGCGAATGCAAGGAAG | GCTTGCACCACTCCAAATCC |
| **hCOL1** | ACTGGCGAAACCTGTATCCG | CCAGTTCTTGGCTGGGATGT |
| **hALP** | GCCAGGATCCTAAAAGGGCA | CATGGCCAGGAAGGTCTCAG |
| **hCTSK** | GGGGGACATGACCAGTGAAG | CAGAGTCTGGGGCTCTACCT |
| **hNFATc1** | GTCCTGGAGATCCCACTCCT | TTCAGGATTCCGGCACAGTC |
| **hDCSTAMP** | CGATTTTTGGGCCCTTGTGG | CACAGGGCCTCTGTTGATGT |
| **hOSCAR** | CCCGCTTGGAGATTTGGACT | AGGACACATCCCGGAAGAGA |
| **hRANK** | ACGGTCTGGAGAGGAAATCAG | TCCAGTAAGGAGGGGTTGGA |

## Supplementary Figures


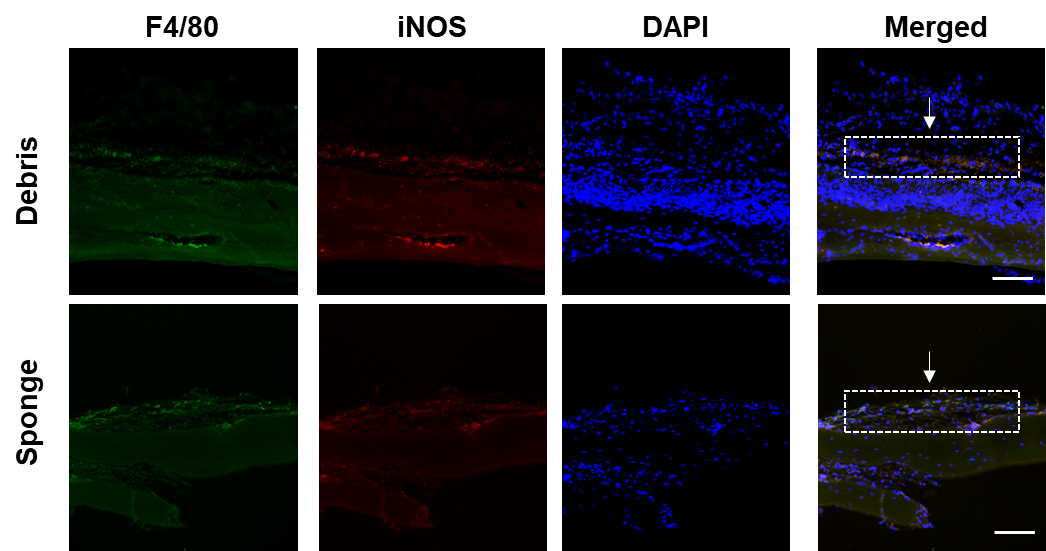


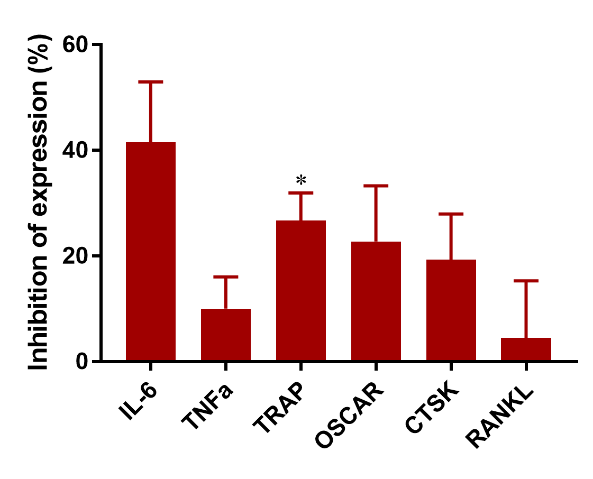
**Supplementary Figure 1.** Detection of monocytic inflammatory cells (F4/80^+^/iNOS^+^) in the cell-infiltrated area. Sections from calvarial tissues of debris-induced osteolysis model (upper panel) and XCL1-soaked sponge model (upper panel) were stained with iNOS (red) and F4/80 (green) antibodies and DAPI (blue). Scale bar is 100 µm. Results demonstrated that inflammatory cells were infiltrated into tissues due to debris implantation and XCL1 administration. Pathological changes were seen with dose of 2μg XCL1 but not 1μg.

**Supplementary Figure 2.** Inhibitory effects of XCL1 antibody treatment on the gene expression of inflammatory and osteoclastogenic factors in granulomatous tissues. Inhibition of gene expression in cells separated from granulomatous tissue surrounding wear debris after treatment with neutralizing antibody. Relative expression of each target gene was normalized based on GAPDH expression. Percentage of inhibition was determined based one values from control mice. Results represent the means of relative expression values ± SEM of 5 mice. * indicates a significant difference as determined by the Student t-test (p ≤ 0.05).


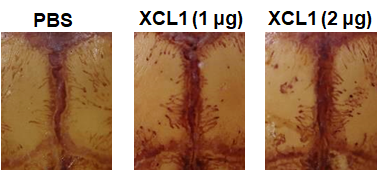


**Supplementary Figure 3.** Representative images for TRAP-stained skulls. Whole skulls were collected, fixed and stained by TRAP staining.


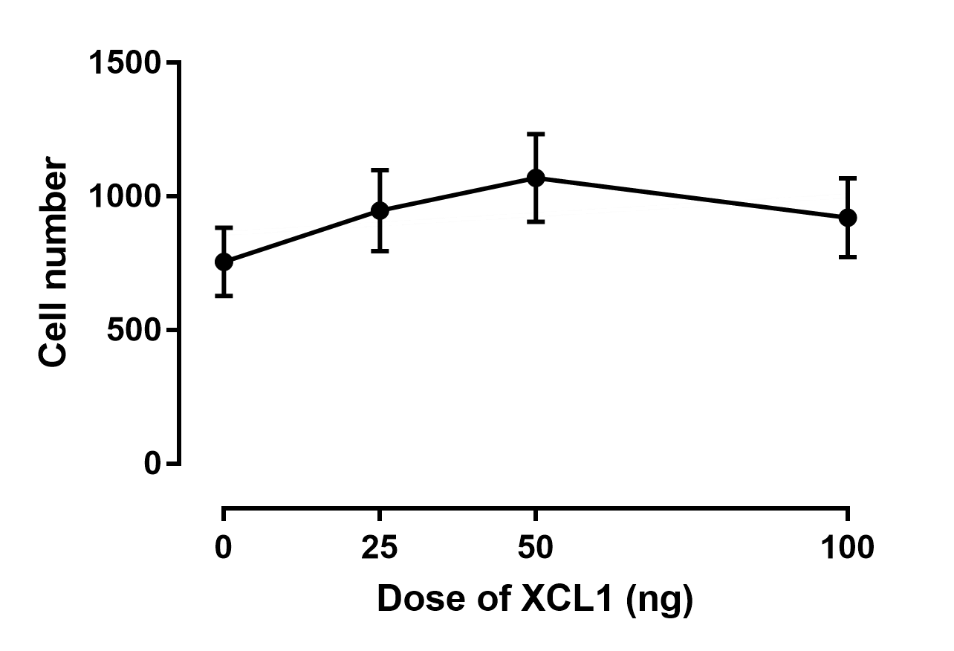


**Supplementary Figure 4.** Synergistic effect of XCL1 on osteoclasts differentiation. Numbers of TRAP-positive cells/well. Human primary monocytes were cultured with growth factors supplemented with recombinant XCL1 at doses of 0, 25, 50, 100 ng/ml for 8 days. Results represent the means of relative expression values ± SEM of triplicates.


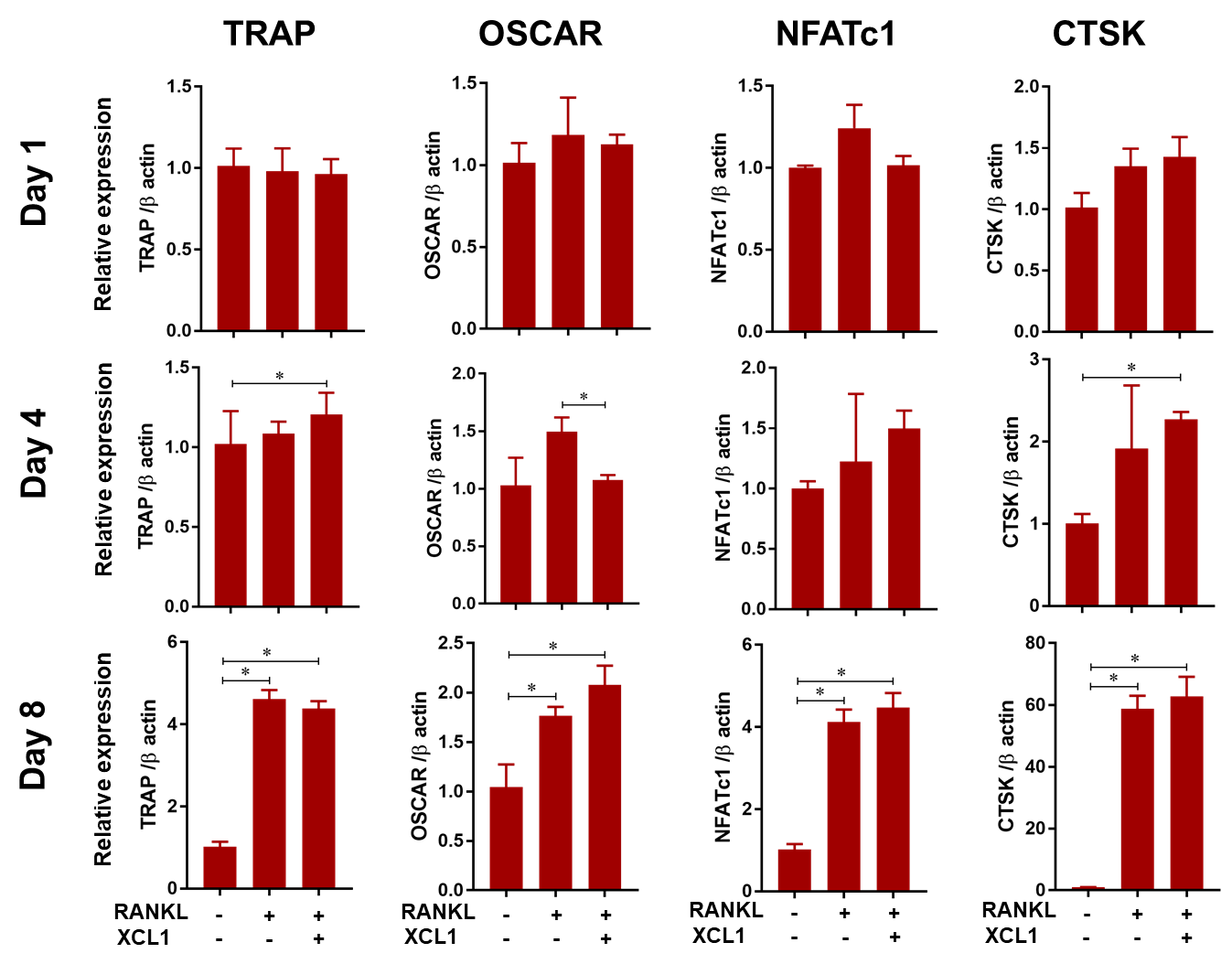


**Supplementary Figure 5.** Gene expressions of osteoclast differentiation markers of human monocytes. Human primary monocytes were separated from the blood samples of healthy donors and collected on day 1, 4 and 8 post-stimulation with either RANKL alone or RANKL plus XCL. The results represent the means ± SEM for triplicates. * indicates a significant difference, as determined by one-way ANOVA, followed by the Tukey's multiple-comparison procedure (p ≤ 0.05).


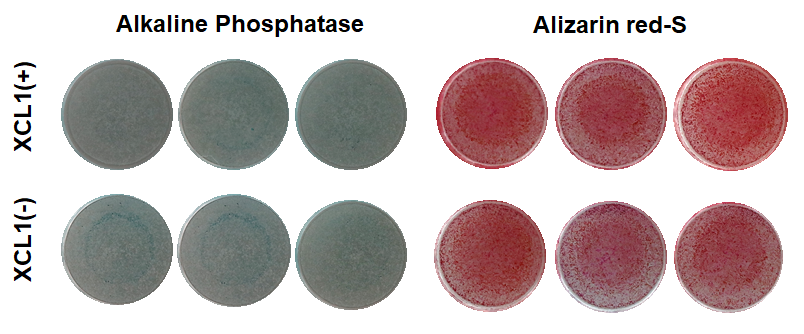


**Supplementary Figure 6.** Alkaline phosphatase (BCIP/NBT) and alizarin red S staining for osteoblasts. Human fetal osteoblasts were cultured in 96-well plate with or without 50 ng/ml of recombinant XCL1 for 21 d. Staining revealed that recombinant XCL1 exhibited no negative effects on the differentiation of osteoblasts.


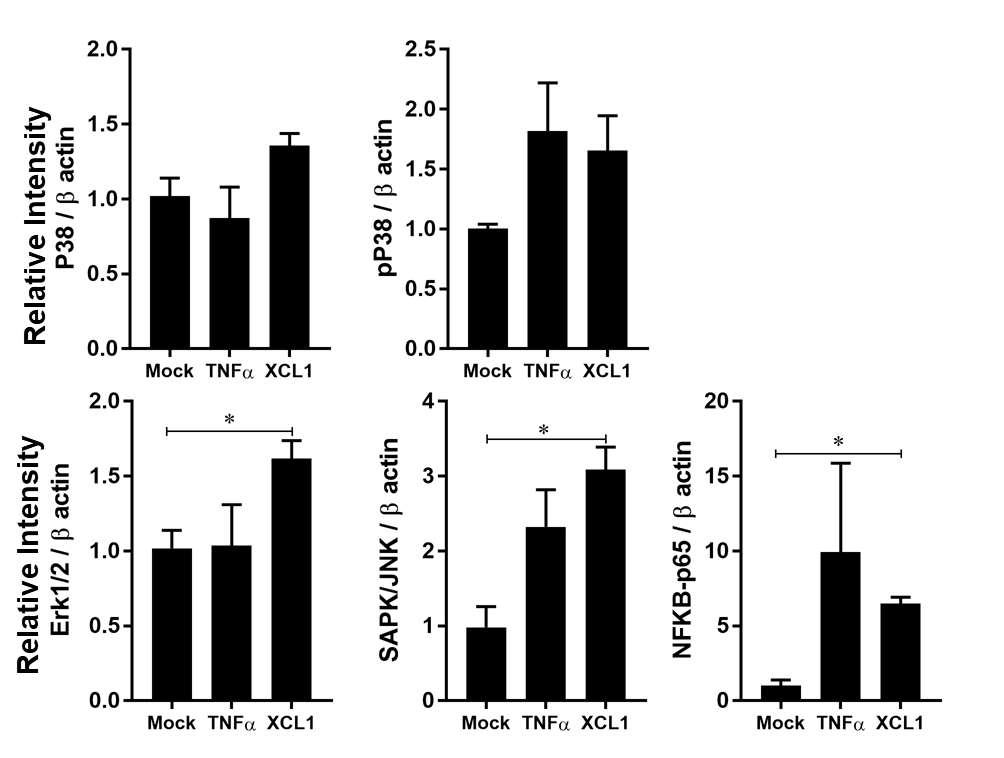


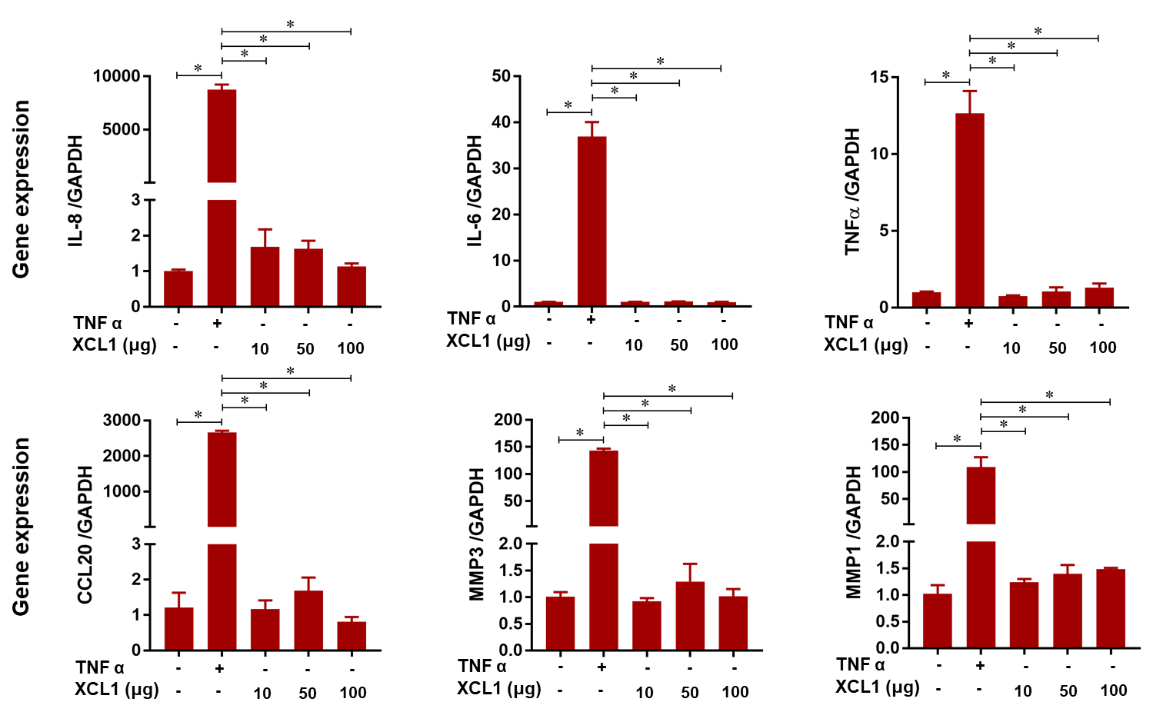
**Supplementary Figure 7.** Quantification of bands detected by Western blotting. Relative intensity of each band was calculated based on bands intensity of target/ β-actin. Results represent the means ± SEM for triplicates. * indicates a significant difference, as determined by one-way ANOVA, followed by the Tukey's multiple-comparison procedure (p ≤ 0.05).

**Supplementary Figure 8.** Effects of recombinant XCL1 on the expression of inflammatory factors in FLS. HFLS from normal healthy human synovial tissues were stimulated with either recombinant TNF-α or XCL1 at concentration of 10, 50 or 100 ng/ml for 48 h. The results represent the means ± SEM for triplicates. * indicates a significant difference, as determined by one-way ANOVA, followed by the Tukey's multiple-comparison procedure (p ≤ 0.05). Results showed that there were no significant increases in the gene expression of IL-8, IL-6, TNFα, CCL20, MMP3 and MMP1 in cells stimulated with XCL1.
